# The ToMenovela – A Photograph-Based Stimulus Set for the Study of Social Cognition with High Ecological Validity

**DOI:** 10.3389/fpsyg.2016.01883

**Published:** 2016-12-02

**Authors:** Maike C. Herbort, Jenny Iseev, Christopher Stolz, Benedict Roeser, Nora Großkopf, Torsten Wüstenberg, Rainer Hellweg, Henrik Walter, Isabel Dziobek, Björn H. Schott

**Affiliations:** ^1^Department of Psychiatry and Psychotherapy, Campus Mitte, Charité Universitätsmedizin BerlinBerlin, Germany; ^2^Humboldt UniversityBerlin, Germany; ^3^Leibniz Institute for NeurobiologyMagdeburg, Germany; ^4^Free University of BerlinBerlin, Germany; ^5^Department of Psychology, Philipps University of MarburgMarburg, Germany; ^6^Otto von Guericke UniversityMagdeburg, Germany; ^7^Center for Behavioral Brain SciencesMagdeburg, Germany

**Keywords:** Theory of Mind, stimulus set, ecological validity, social cognition, photographs, empathy, emotions

## Abstract

We present the ToMenovela, a stimulus set that has been developed to provide a set of normatively rated socio-emotional stimuli showing varying amount of characters in emotionally laden interactions for experimental investigations of (i) cognitive and (ii) affective Theory of Mind (ToM), (iii) emotional reactivity, and (iv) complex emotion judgment with respect to Ekman’s basic emotions (happiness, anger, disgust, fear, sadness, surprise, Ekman and Friesen, 1975). Stimuli were generated with focus on ecological validity and consist of 190 scenes depicting daily-life situations. Two or more of eight main characters with distinct biographies and personalities are depicted on each scene picture. To obtain an initial evaluation of the stimulus set and to pave the way for future studies in clinical populations, normative data on each stimulus of the set was obtained from a sample of 61 neurologically and psychiatrically healthy participants (31 female, 30 male; mean age 26.74 ± 5.84), including a visual analog scale rating of Ekman’s basic emotions (happiness, anger, disgust, fear, sadness, surprise) and free-text descriptions of the content of each scene. The ToMenovela is being developed to provide standardized material of social scenes that are available to researchers in the study of social cognition. It should facilitate experimental control while keeping ecological validity high.

## Introduction

Recent years have seen a steep increase in behavioral and brain imaging research of human social cognition. Defining, differentiating and operationalizing cognitive and emotional subprocesses of social cognition such as empathy, Theory of Mind (ToM), and emotion recognition, have attracted increasing interest from psychologists and neuroscientists. Two related, but yet separable constructs have been employed by researchers to describe the cognitive processes that may enable humans to understand others’ cognitive and affective states – empathy and ToM. While ToM describes the ability to understand and predict another’s mental states, intentions, or beliefs, empathy as a psychological construct rather describes the phenomenon to share other people’s affective states, which is likely to form the basis for social emotions like guilt or compassion. Hein and Singer (2008) explicitly distinguish empathy from “cognitive perspective taking as the ability to understand intentions, desires, beliefs of another person, resulting from (cognitively) reasoning about the other’s state”, a concept that can be called “cognitive empathy”, whereas the classical definition could be referred to as “affective empathy.” The related concept of mentalizing (Frith and Frith, 2006) has been defined as “the process by which we make inferences about mental states” and comprises an immediate recognition and understanding of emotional states, also via cognitive inference. A triple-dissociation of the ToM/empathy complex suggested by Walter (2012) divides the ToM concept into three separable cognitive mechanisms: *Cognitive ToM* comprises the ability of an individual to mentalize about cognitive states of others, *Affective ToM* – or *Cognitive Empathy* – is defined as an individual’s ability to cognitively reflect on affective states of others, and *Affective Empathy* is characterized by the induction of others’ affective states in the perceiving individual.

Numerous experimental paradigms have been developed to formalize the ToM construct in a way that allows researchers to assess both behavioral manifestations and neural underpinnings of ToM-related cognitive mechanisms. These include the well-known *False Belief Task* (initially developed by Wimmer and Perner, 1983), a paradigm commonly used in developmental research, and the related *Sally-Anne Tasks* (Baron-Cohen et al., 1985), which have been employed to demonstrate ToM deficits in children with Down’s Syndrome and Asperger’s Syndrome. A different approach to the experimental assessment of ToM and empathy was introduced with the publication of the *Reading the Mind in the Eyes Task* (*RMET*; Baron-Cohen et al., 1997), in which participants have to assign mental states to static pictures of eye regions. Notably, comparisons of the behavioral performance in different ToM tasks have yielded poor correlations (Ahmed and Miller, 2013).

Despite this lack of correlation, the cognitive processes tested by the presently available tasks do most likely all contribute to enabling ToM in real-life social situations. It is conceivable that, in the real world, people rely on highly multimodal information when engaging in social cognitive tasks, and different individuals are therefore likely to potentially employ distinct strategies during social cognition. Achim et al. (2013) have proposed the *Eight Sources of Information Framework* (8-SIF) as a theoretical framework to analyze mentalizing tasks with respect to the information participants can use for task performance. It consists of a 2*2 matrix, with the axes reflecting the temporal characteristics of information [*immediate* (I), with the subcategories “*linguistic*” and “*perceptual*”, vs. *stored* (S), with the subcategories “*general*” and “*source-specific*”] and *agent*-related versus *context*-related information. The authors suggest that the multimodal nature of information described in the 8-SIF framework is best met by more naturalistic – or ecologically valid – paradigms or stimuli.

The need for ecologically valid stimulus material has been recognized in cognitive neuroscience, and several stimulus sets of various categories have been developed for this purpose. For example, a number of photograph-based sets of object stimuli have been developed as an alternative for the commonly used Snodgrass pictures, line drawings of common objects (Snodgrass and Vanderwart, 1980). These include the *Amsterdam Library of Object Images* (ALOI; Geusebroek et al., 2005) or the *Bank of Standardized Stimuli* (BOSS; Brodeur et al., 2014)^1^. The importance of examining ecologically valid information is well-established in the field of visual perception research (Kayser et al., 2004), but only few ecologically valid stimulus sets applicable to emotion processing and social cognition have been published so far. A notable exception is the *International Affective Picture System* (IAPS; Lang et al., 2008), which contains images of different degrees of emotional valence and arousal, including highly aversive images of accidents and mutilation.

Based on the IAPS stimuli, the MET (*Multifaceted Empathy Test*; Dziobek et al., 2008) has been developed to study both affective ToM as well as affective empathy. In this photograph-based stimulus set, human beings are depicted in various emotional situations and participants are asked to infer the mental states of the persons depicted (affective ToM) and to indicate the level of own emotional involvement when perceiving or evaluating the scenes (affective empathy). The MET has been extensively validated by experts and is therefore suitable for assessing response accuracy in social cognitive tasks. One potential limitation of the MET is that the images are based on IAPS stimuli, which are –to a large extent– not representative for daily-life situations.

With a strong focus on ecological validity, Dziobek et al. (2006) have developed the MASC (*Movie for the Assessment of Social Cognition*). The stimulus set consists of a 15-min video showing four main characters at a dinner party. In 46 breaks, subjects have to answer questions on the feelings, thoughts, and intentions of the characters. The task shows rather high ecological validity, but its design as a movie with a fixed location and a small number of protagonists limit its use particularly in neuroimaging studies that require precise trial timings and appropriate baseline conditions. In neuroimaging studies of ToM and empathy, it is also important to employ appropriate controls, both at the task level (e.g., first-person perspective versus “pure” ToM) and at the item level (e.g., different degrees of task difficulty or emotional salience and valence), preferably using the same stimulus material. Schnell and Walter have developed a task that allows one to distinguish first-person and third-person perspective during emotional and cognitive/visual-perceptual processing (Schnell et al., 2011; Walter et al., 2011). The stimulus set consists of cartoon stories that are usable as false-belief tasks, but have been designed in a way that suitable first-person perspective control questions can also be applied to all stories. Cartoon stories consisting of three sequentially presented pictures are shown, and participants are instructed to either count the number of animate objects (self-cognitive), to state whether the protagonist can see more or less animate objects than in the previous picture (third-person cognitive), whether they feel better or worse than during the picture presented before (first-person affective), or whether the protagonist feels better or worse than during the previous picture (other-affective). Notably, that stimulus set is devoid of any direct indicators of the protagonists’ affective states, like expressive facial elements.

**FIGURE 1:**
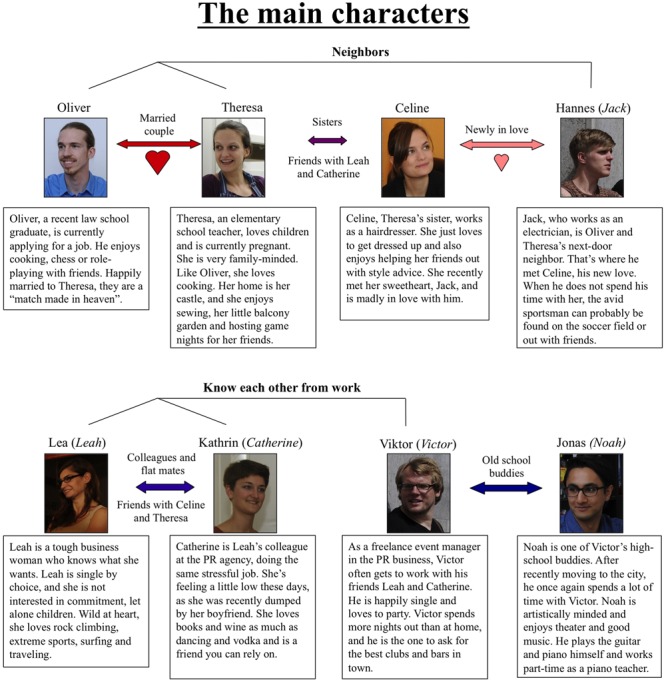
**Description of the main characters and interpersonal relations within the group.** The names and biographies shown here were used in our evaluation study, but future researchers should be readily able to adapt them to their needs. Suggested English names in italics are suggestions from the authors to replace the German names used during evaluation.

Here, we present a stimulus set (*The ToMenovela*) that was specifically designed to combine the high ecological validity of the MASC and the MET with the applicability of first-person control tasks as in the cartoon task by Schnell and Walter. We chose to base the task on photographs rather than movies, in order to make it more suitable for event-related fMRI and EEG studies. To achieve high ecological validity, we set up a fictional circle of eight friends (four male and four female; see **Figure 1**) and designed a background story that contains biographies and personalities of each protagonist as well as the relationships between the characters. Each of the characters possesses stable characteristics (traits) that are distinct from one another (e.g., homely, outgoing, artistic, etc.). Based on this social arrangement, we scripted a series of scenes that would be comprehensible from a single still photograph. We aimed to balance the scenes with respect to location (indoor vs. outdoor) and appearance of the characters (each scene depicts at least two of the protagonists). After selection of the suitable stimuli, we collected normative data on the stimulus set in a cohort of 61 healthy study participants (31 women, 30 men), in order to obtain normative data with respect to content, emotional salience and valence, as well as cognitive and affective ToM. Because emotion recognition constitutes an important facet of human social cognition, the scenes were designed to Ekman’s basic emotions (happiness, anger, disgust, fear, sadness, surprise; Ekman et al., 1972; Ekman and Friesen, 1975, 1978) to a various degree, and the evaluation contained specific questions testing for emotion recognition (see Methods section for details). One important reason for including Ekman’s emotions was the potential for future clinical applications: Emotion recognition and cognitive ToM show parallel deficits in certain neuropsychiatric disorders like schizophrenia (Sparks et al., 2010; Barbato et al., 2015) or temporal lobe epilepsy (Amlerova et al., 2014), but may be differentially affected in other conditions like Alzheimer’s disease and frontotemporal dementia (Gregory et al., 2002; Freedman et al., 2013). Therefore, the inclusion of Ekman’s emotions may be useful for future clinical applications.

As will be outlined in the following sections, the ToMenovela has several potential advantages for future studies of human social cognition:

(1) With respect to ecological validity, the use of a defined group of protagonists may induce a sense of familiarity, thereby accounting for the fact that most social interactions in daily life occur with individuals with whom humans are at least to some extent familiar.
(2) Also for the purpose of high ecological validity, scenes were designed to differ in their emotional salience and valence, but we avoided extreme emotional situations, in order to match the content of the scenes with the daily-life experience of the likely study participants.
(3) By using photographs, the stimulus set is highly suitable for event-related neuroimaging studies.
(4) Finally, beyond social cognition, the stimuli may also be suitable for studies of other cognitive processes like higher-level vision, memory, or face and scene processing (Zweynert et al., 2011; Hofstetter et al., 2012; Rossion et al., 2012).

## Materials and Methods

**FIGURE 2:**
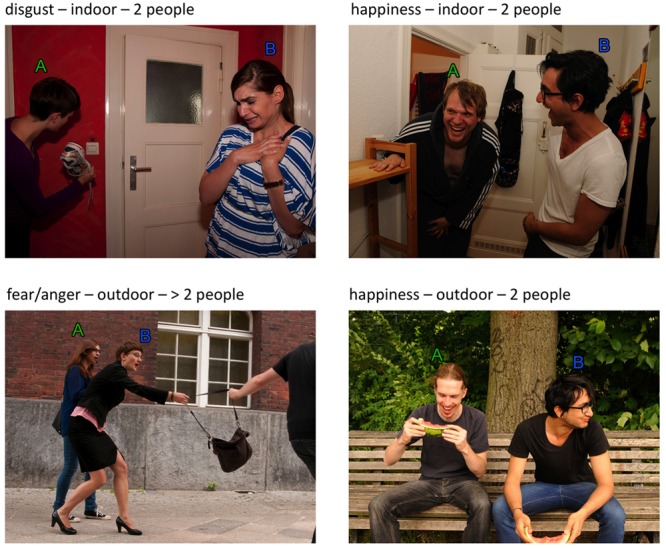
**Four example pictures.** The pictures shown here were generated along with the actual stimulus set, but excluded for technical reasons. They are nevertheless representative for our stimulus set and should be used in publications.

In order to generate a stimulus set of pictures depicting daily-life social interactions for use in future studies of social cognition, we scripted a total of 220 distinct daily-life scenes, 193 of which were subsequently staged and photographed (see **Figure 2** for example scenes). Because we aimed to generate stimuli that would be particularly suitable for neuroimaging studies, we opted for photographs rather than video clips. Two scenes were excluded due to technical problems, and one due to ambiguous evaluation results, resulting in a final set of 190 scenes.

In a subsequent validation study, each scene was rated with respect to principal content, cognitive and affective first- and third-person perspective, emotional valence along six basic emotions (happiness, anger, disgust, fear, sadness, surprise; Ekman et al., 1972; Ekman and Friesen, 1975, 1978). Those ratings were complemented by two free-text open questions, and the response data will be reported in a future publication.

### Generation of the Stimulus Material

#### Script

We first developed an initial sketch of eight distinct human characters that constitute a circle of friends with diverse relationships (a long-term married couple, a new romantic relationship, two sisters, colleagues, high school friends, the “new guy in town”, etc.). **Figure 1** describes the biography and personality traits of the main characters and the interpersonal relations within the group.

We next scripted a total of 220 scenes, each of which was to depict at least two of the eight main characters. Each scene was constructed with respect to general content, basic emotions (happiness, anger, disgust, fear, sadness, surprise), dramatic setting, characters displayed, requisites, and location. The scripts also included mindsets of the different protagonists instructing the actors to feel and express specific emotions (for example scripts, see Supplementary Tables S1A,B). When scripting the scenes, we aimed to balance the appearance of the eight main characters, basic emotions and location (indoor vs. outdoor). Due to external conditions during the shooting of the scenes (e.g., sicknesses of actors or unexpected weather changes), some scenes deviated in details from their original script.

#### Team

We recruited eight professional and semi-professional actors as main cast and, depending on the specific scene, additional experienced lay actors. The cast for the main characters and reoccurring background actors were recruited in early 2013. The final ensemble consisted of two professionally educated actors and six amateurs with previous stage experience (drama and/or music). The actors were known to each other prior to the shootings and specifically selected based on their certain style and personality, although it should be noted that their actual biography and personality differ from that of the fictional characters described here. All actors gave written informed consent for the use of the resulting photographs for research purposes.

All main actors were familiarized with their respective character by authors MCH, a trained psychologist, and BR who holds a B.A. in theater studies and has extensive previous experience in directing. MCH and BR also directed and supervised the shootings of all scenes.

Photographs were acquired and processed by Sven Reichelt^2^, a photographer with extensive previous experience in portrait photography.

#### Shootings

To ensure a continuous look and feel of each character, clothes, accessories, and make-up were obtained from a previously assembled pool of equipment prior to the beginning of the shootings. Each shooting session was carefully prepared in terms of location, equipment, clothes, make-up, and look. Depending on the complexity of the scene and external conditions (e.g., availability of the actors, weather conditions at the time of shooting), between four and 22 different scenes were shot on one day. All shootings took place in Berlin, Germany, between May 4th, 2013 and July 20th, 2013. Because the scenario is intended to take place in an unnamed major city in an unspecified country in Europe (possibly also North America or Australasia), we aimed to minimize recognizable German writing and strictly avoided any iconic buildings (e.g., the Brandenburg Gate or the Emperor William Memorial Church) in the pictures.

Photographs were taken using a Nikon D300s digital SLR camera with a sensor size of 23.6 mm × 15.8 mm and a resolution of 12.3 megapixel (4352 × 2868). All pictures were taken in sRGB color mode. Depending on the requirements posed by the scene, either a AF-S Nikkor 16-85 mm1:3.5 – 5.6G ED medium-angle lens or a Sigma 10–20 mm F 4.0 – 5.6 EX DC HSM wide angle lens were used. If necessary, two Nikon SB900 were used as flash.

### Post-processing and Picture Selection Procedure

We used a multi-level picture selection and processing procedure to obtain a final set of images that best represented the intended social interactions and emotional valance.

Pictures were first screened for technical, compositional, and photographic aspects. All approximately 10 000 pictures were screened with respect to sharpness, lighting conditions or unintended facial expressions and with regard to the final aspect ratio. To this end, the photographer and the first author selected between one and eight pictures per scene for post-processing. Post-processing of the pictures was done using PhotoShop (Adobe, San José, CA, USA) and the open source image manipulation software GIMP^3^. Camera RAW images were adjusted for brightness, contrast and color, and converted into JPG format. All images were clipped horizontally to set the horizontal to vertical aspect ratio to 4:3. When necessary (e.g., due to distracting content outside the focus of the picture), images were clipped further, keeping the aspect ratio.

A resulting set of 555 pictures belonging to 191 scenes was presented to five raters who had not been involved in the initial shootings and did not know the actors personally (authors CS and NG, prior to their further participation in normative data collection and/or data analysis; and one other man and two other women). They were asked to answer two questions on a 5-point Likert scale.

(1) How clearly can you identify the depicted situation/interaction? [*clarity*; “completely ambiguous or random” to “completely unambiguous”]
(2) How clearly can you identify (any) emotions in the scene? [*emotion*; “not at all” to “very clearly”]

Based on the raters’ responses, weighted sum scores were calculated (clarity ^∗^ 3 + emotion), and the pictures with the highest sum scores were selected for the final picture set. The aim of this pre-rating procedure was to have only one picture per scene with the highest possible rating clarity. It left 46 scenes for which two or more pictures had equally high scores. The pictures in question were inspected by the first and last authors, and the final image was selected based on consensus. The resulting final set of 191 unique images was used in the validation study. **Figure 2** depicts four example images [*Note:* The pictures displayed here are not part of the actual stimulus set and may be used for illustrative purposes in publications].

### Normative Data Collection Study

The evaluation of the final stimulus set of 191 pictures was performed using a computer-based rating procedure and was carried out in Berlin and Magdeburg, Germany, from December 2014 to November 2015.

#### Participants

Sixty-one participants of the validation study (31 women, 30 men) were recruited via advertisements, through various academic mailing lists, and by contacting former participants of earlier experiments done by the authors. A total of 41 participants (26 female) were recruited and tested in Berlin, and 20 participants (five female) performed the task in Magdeburg. Detailed demographic data of the study cohort are displayed in **Table 1**. People interested in participating were first informed about the evaluation process via e-mail and were asked to answer to a set of psychological questionnaires at home, including a general health questionnaire and the Structured Clinical Interview for DSM-IV, (First et al., 1996, 1997; Saß et al., 2003) Section II (SCID-II) screening questionnaire. Participants were interviewed for present or past DSM-IV psychiatric disorders using a SCID-I-based screening questionnaire and the appropriate SCID-I modules when applicable. Clinical interviews were performed by the first author under supervision of the last author, who is a board-certified psychiatrist. Exclusion criteria were insufficient knowledge of the German language, a history of head trauma, neurological illness, bipolar disorder, schizophrenia or substance use disorder, and the use of centrally acting medication. Participants with above-cut-off values in the SCID-II questionnaire were interviewed according to the SCID-II manual by the first author, and a potential clinically relevant diagnosis led to exclusion from the study. All participants gave written informed consent prior to the participation in the study in accordance with the Declaration of Helsinki and received financial reimbursement. The study was approved by the Ethics Committee of the University of Magdeburg, Faculty of Medicine.

**Table 1:** Demographic and psychometric parameters.

	Male (*n* = 30)		Female (*n* = 31)		Statistics
	Parameter	distribution	Parameter	distribution	
Age	ø = 27.10 (±4.54); min = 19, max = 40		ø = 26.39 (±6.92); min = 19, max = 49		*t*_59_ = -0.474, n.s.
Smoking	Yes = 3; Never = 22; Former or occasional = 5		Yes = 1; Never = 22; Former or occasional = 8		Fisher’s exact test: *F* = 1.61, n.s.
Education	ø = 17.97 (±2.83); min = 12, max = 22		ø = 17.15 (±2.69); min = 12.5, max = 22		*t*_59_ = -1.163, n.s.
LPS (PR subtest 3+4)	ø = 91.21 (±7.35)	SW = 0.817, *p* < 0.001	89.38 (±9.43)	SW = 0.81, *p* < 0.001	*U* = 432, *Z* = 0.714, n.s.
MWT-B (IQ)	ø = 100.87 (±5.53)	SW = 0.979, n.s.	100.00 (±7.19)	SW = 0.833, *p* < 0.001	*U* = 394, *Z* = 0.823, n.s.
BDI (sum)	ø = 3.90 (±3.44)	SW = 0.877, *p* < 0.05	2.42 (±2.78)	SW = 0.803, *p* < 0.001	*U* = 344.5, *Z* = 0.823, n.s.
STAI-trait (PR)	ø = 50.03 (±29.88)	SW = 0.934, n.s.	45.45 (±25.55)	SW = 0.96, n.s.	*t*_59_ = -0.644, n.s.
STAXI					
Subscale State Anger (normal range: 10–40)	ø = 10.77 (±1.61)	SW = 0.569, *p* < 0.001	11.16 (±1.90)	SW = 0.639, *p* < 0.001	*U* = 388, *Z* = 0.722, n.s.
Subscale Trait Anger (normal range: 5–20)	ø = 7.93 (±4.03)	SW = 0.629, *p* < 0.001	7.03 (±1.78)	SW = 0.85, *p* < 0.05	*U* = 442, *Z* = 0.659, n.s.
Subscale Anger Temperament (normal range: 5–20)	ø = 7.90 (±2.19)	SW = 0.922, *p* < 0.05	8.55 (±2.77)	SW = 0.877, *p* < 0.05	*U* = 413, *Z* = 0.495, n.s.
Subscale Anger Reaction (PR)	ø = 35.70 (±26.73)	SW = 0.907, *p* < 0.05	34.90 (±25.03)	SW = 0.897, *p* < 0.05	*U* = 442, *Z* = 0.714, n.s.
Subscale Anger-in (PR)	ø = 38.70 (±35.92)	SW = 0.834, *p* < 0.001	22.00 (±20.47)	SW = 0.872, *p* < 0.05	*U* = 371, Z = 1.05, n.s.
Subscale Anger-out (PR)	ø = 50.23 (±17.91)	SW = 0.962, n.s.	48.06 (±18.83)	SW = 0.912, *p* < 0.05	*U* = 414, *Z* = 0.584, n.s.
Subscale Anger Control (PR)	ø = 49.93 (±23.71)	SW = 0.958, n.s.	52.26 (±26.05)	SW = 0.939, n.s.	*t*_59_ = -0.364, n.s.
BIS (sum)	ø = 59.97 (±8.43)	SW = 0.94, n.s.	59.71 (±9.94)	SW = 0.977, n.s.	*t*_59_ = -0.109, n.s.
ADHS (sum)	3.03 (±3,15)	SW = 0.76, *p* < 0.001	2.81 (±3.59)	SW = 0.749, *p* < 0.001	*U* = 406, *Z* = 0.483, n.s.
AQ (sum)	17.62 (±7.02)	SW = 0.955, n.s.	12.90 (±4.99)	SW = 0.945, n.s.	*t*_59_ = -2.985, *p* < 0.05
SPF – Fantasy (*M* = 100, *SD* = 10)	93.24 (±8.16)	SW = 0.965, n.s.	101.40 (±8.62)	SW = 0.909, *p* < 0.05	*t*_59_ = 3.731, *p* < 0.001
SPF – Empathic concern (*M* = 100, *SD* = 10)	98.69 (±6.70)	SW = 0.951, n.s.	104.83 (±6.84)	SW = 0.938, n.s.	*t*_59_ = 3.485, *p* < 0.001
SPF – Perspective taking (*M* = 100, *SD* = 10)	102.59 (±9.07)	SW = 0.941, n.s.	103.73 (±7.85)	SW = 0.95, n.s.	*t*_59_ = 0.520, n.s.
SPF – Personal distress (*MW* = 100, *SD* = 10)	93.34 (±6.29)	SW = 0.934, n.s.	97.63 (±7.43)	SW = 0.969, n.s.	*t*_59_ = 2.389, *p* < 0.05
SPF – Score (*M* = 100, *SD* = 10)	98.17 (±5.67)	SW = 0.979, n.s.	103.40 (±5.98)	SW = 0.946, n.s.	*t*_59_ = 3.44, *p* < 0.001

*Demographic information and psychometric measures are displayed separately for male and female participants. PR, percentile rank; LPS, Leistungsprüfsystem – subtests 3 + 4; MWT-B, Mehrfachwahl-Wortschatz-Intelligenztest form B; BDI, Becks Depression Inventory (Beck et al., 1961); STAI, State-Trait Anxiety Inventory (Laux et al., 1981); STAXI, State-Trait Anger Expression Inventory (Schwenkmezger et al., 1992); BIS, Barratt Impulsiveness Scale (Patton et al., 1995); ADHS, ADHS-Diagnose-Checkliste (Rösler et al., 2004); AQ Autism Spectrum Quotient (Dammann, 2002); SPF, Saarbrücker Persönlichkeitsfragebogen. Standard deviations are given in parentheses; *T*-tests were calculated 2-tailed. In case of normal distribution, *t*-tests were calculated. All scales met the Levene-Test. In case of not normally distributed, Mann–Whitney-*U* (*U*) and Kolmogorov–Smirnov-*Z* (*Z*) were calculated.*

#### Schedule

Participants received the biographical chart (**Figure 1**) to familiarize them with the characters and their backgrounds and relationships. This was done for the purpose of further increasing ecological validity, as most daily-life social interactions occur with familiar individuals. Seven days (±2 days) after receiving the chart, participants were scheduled for the actual rating procedure. Due to the length of the procedure, the experiment was split into three experimental sessions that were performed within three to seven days. At the beginning of the study, participants were asked to provide their individual impression of the eight protagonists in written form and to fill in a paper–pencil two-alternative forced-choice quiz designed to ensure that they were sufficiently familiar with the characters (for example questions, see Supplementary Table S2; the complete quiz is available along with the stimulus set).

#### Experimental Paradigm

The actual experiment started with a standardized instruction provided by the experimenter (author MCH, JI, or NG). The participants were explained that they would be presented with scenes depicting the eight characters in various daily-life situations in a total of 191 pictures. The pictures would have no chronological timeline and were to be considered independently from each other.

Pictures were presented on a computer screen (resolution 1600×1200 or 1920×1080) at a resolution of 700 × 525 pixels, together with a set of task instructions presented sequentially. The same rating tasks were performed for each of the images:

(1) Description of the content and one’s own behavioral reaction in free-text format.
(2) Emotional salience and valence on seven dimensional scales:
  (a) one scale assessing emotional salience (*first-person affective*)
  (b) valence ratings across the six basic emotions according to Ekman
(3) Affective ToM (*third-person affective*): This condition intended to operationalize affective ToM and to some degrees also emotion recognition. Two of the characters depicted were marked with “A” and “B”, and subjects responded to the question which person was feeling better on the scene depicted (multiple-choice answer format: A, B, both equally).
(4) Cognitive ToM (*third-person cognitive*): In analogy to the affective ToM question, two characters were marked with “A” and “B”, and participants were asked to indicate which of the two characters could see more people in the scene (multiple-choice answer format: A, B, both equally).

The affective and cognitive ToM tasks were designed to closely match the cognitive ToM tasks used in the previously described cartoon-based ToM paradigm developed by Schnell et al. (2011) and Walter et al. (2011). Because single pictures rather than sequences were presented, we opted for the use of a comparative task between two protagonists (instead of the within-subject across-sequence rating employed by Schnell and Walter). Also to match the task by Schnell and Walter, the cognitive ToM task required visual perspective taking (original task: number of animate objects seen by the protagonist; present task: number of human beings seen by the two protagonists).

Because all ratings were performed by lay participants – that is, no data from either experts or clinical populations were collected – they represent normative data rather than accuracy scores at this point. Expert ratings of the ToMenovela are, however, currently in preparation. While absolute accuracy scores cannot be conclusively determined from the ratings performed so far, our normative data do provide information with respect to ambiguity, which reflect in part difficulty of an item. Thus, researchers may use this information to generate subsets of stimuli sets with different degrees of ambiguity and thus varying difficulty.

All task instructions, along with the corresponding response options and the purpose of each question are summarized in **Table 2**. The task was self-paced, and participants could interrupt the rating procedure at any time to ensure that they would remain alert for the entire experiment. **Supplementary Figure S1** depicts an example trial. The software used for the rating procedure was programmed in Java (Oracle, Redwood City, CA, USA) by author CS and is available from the authors upon request.

**Table 2:** Task instructions.

Task	Answer options	Purpose
Describe the scene in your own words.	Free text format	Recognition of the content of the scene
Does person A or person B feel better?	Check-boxes: Person A Person B Both alike	Assessment of affective ToM (i.e., cognitive empathy; see Walter, 2012) Emotion recognition
How much do you feel affected by the picture?	Visual analog scale, designed as a slider, ranging from “not at all” to “very much”	Affective empathy Emotional recativity
Who can see more people?	Check-boxes: Person A Person B Both equally	Cognitive ToM (in analogy to Schnell et al., 2011) Visual perspective taking
How strongly do you recognize the following emotions in the scene: Happiness, Anger, Disgust, Fear, Sadness, Surprise	Six sequentially presented visual analog scales, designed as a slider, ranging from “not at all” to “very much”	Emotion recognition Emotional reactivity
What would you do if you were to enter the scene?	Free text format	Social competence Approach/avoidance behavior

#### Psychometric Questionnaires and Correlations with Stimulus Rating Data

To ensure that participants of the rating procedure were psychopathologically healthy, all participants received a set of well-established psychometric questionnaires, including the Beck Depression Inventory (BDI, Hautzinger et al., 1994), questions 21–40 from the State-Trait Anxiety Inventory (STAI-trait, Spielberger and Lushene, 1966; Laux et al., 1981), the State-Trait Anger Expression Inventory (STAXI, Schwenkmezger et al., 1992), the Barratt Impulsiveness Scale (BIS, Preuss et al., 2003) and an attention deficit hyperactivity disorder checklist (ADHS-CL, adapted on Rösler et al., 2004). The Autism Questionnaire by Baron-Cohen (AQ, Baron-Cohen et al., 2001) and the Saarbrücker Persönlichkeitsfragebogen (SPF, Paulus, 2009) were administered to the participants in an online-based follow-up survey in autumn 2015. As measures of cognitive functions, the Leistungsprüfsystem (LPS, Horn, 1983) and the Mehrfachwahl-Wortschatz-Intelligenztest (MWT, Lehrl, 2005) were obtained, either prior or after the evaluation session.

To allow for correlational analyses of stimulus ratings and psychometric data, we computed numeric measures that reflected individuals’ “typical” response behavior across the stimuli. Specifically, we computed a measure of decisiveness in the third-person affective and third-person cognitive conditions ([OA_A_ + OA_B_]/OA_both_), a measure of the tendency to make non-standard responses (i.e., the tendency to chose a response not chosen by the majority of the participants), as well as the mean emotion recognition ratings for the Ekman emotions across scenes. These measures were correlated with the SPF subscales and with the AQ, employing non-parametric Spearman correlations and robust Shepherd’s Pi correlations that include an outlier exclusion based on the bootstrapped Mahalanobis distance (Schwarzkopf et al., 2012). All correlations were computed for 59 participants, due to missing SPF and AQ data from one male and one female participant.

## Results

### Stimuli

As a result of the rating procedure, one image (#164) had to be excluded due to ambiguous interpretation by the raters, leaving a total of 190 images in the stimulus set. Supplementary Table S3 displays the basic characteristics of the images.

### Demographic and Psychometric Results

The demographics and psychometric data of the study cohort are presented in **Table 1**, separated by gender. Women and men in our sample did not differ with respect to age, education, and cognitive measures (assessed with LPS and MWT). There were also no significant differences regarding depressive symptoms (BDI), trait anxiety (STAI), anger (STAXI), or impulsivity (BIS-11). Fisher’s exact Test yielded no difference [*F* = 1.607, *p* = 0.460] with respect to smoking status.

Across the study sample, autism- and empathy-related questionnaires revealed scores in line with previous normative data of the AQ (Baron-Cohen et al., 2001) and the SPF.^4^ In both questionnaires, we observed gender differences in the expected directions: male participants had higher mean scores in the AQ (*t*_59_ = -2.985, *p* = 0.004), while in the SPF, male participants had lower scores on the subscales fantasy (*t*_59_ = 3.731, *p* < 0.001), empathic concern (*t*_59_= 3.485, *p* < 0.001), personal distress (*t*_59_= 2.389, *p* = 0.02), and the overall score (*t*_59_= 3.44, *p* < 0.001), but no significant difference in perspective taking (*t*_59_= 5.20, *p* < 0.605).

### Behavioral Results

The results from free-text ratings (descriptions of each scene’s content and one’s own behavioral reactions) are not part of the present work and will be reported separately.

#### Ratings of Emotional Salience and Valence

**FIGURE 3:**
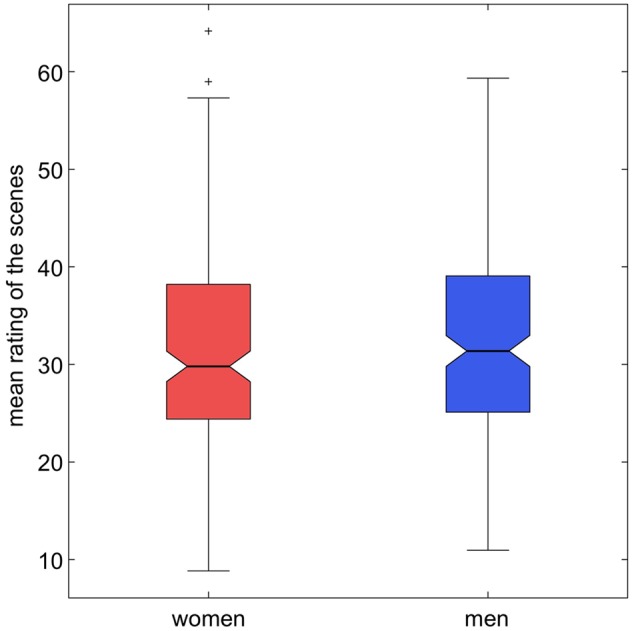
**Mean scores of first-person affective condition “How much do you feel affected by the picture?”, separated by gender.** Box plots depict medians, 25 percent quantiles and outliers.

**Figure 3** depicts the result of the affective salience rating, separated by gender. When asked “How much do you feel affected by the picture” and responding on a slider comparable to a Likert scale, participants gave the scenes a median rating of approximately 30 percent (women: 29.8; men: 31.4), with a broad range from approximately 10 to 60 percent (women: 8.8 – 64.2; men: 11.0 – 59.3). We provide detailed descriptive statistics of the affective salience ratings (mean, median, mode, standard deviation, skewness, standard deviation of skewness, curtosis, standard deviation of curtosis) for each scene as along with the stimulus set.

**FIGURE 4:**
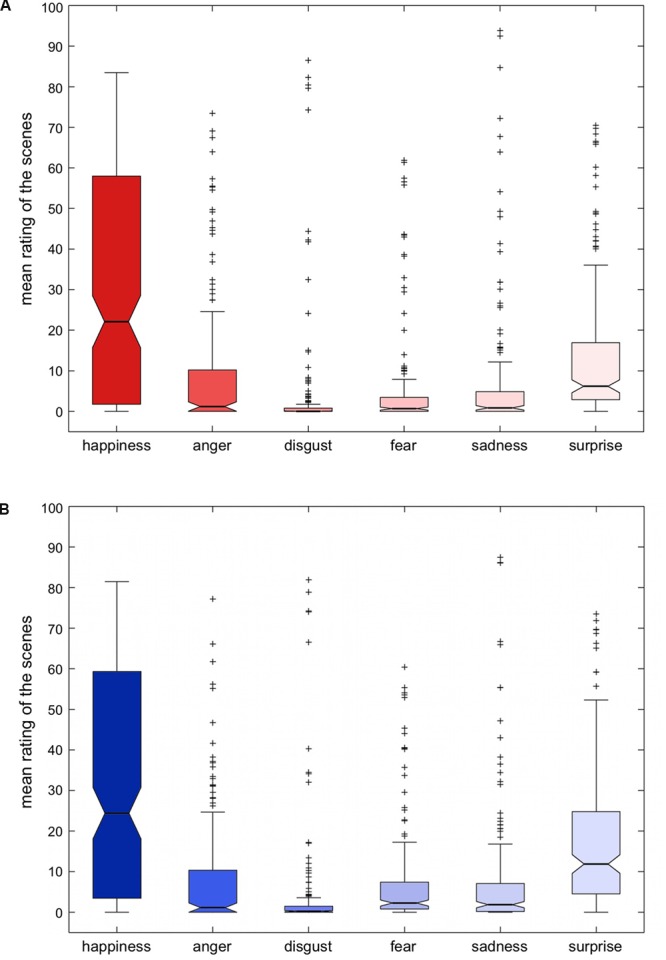
**Mean scores of emotional valence, separated by gender.** Box plots depict medians, 25 percent quantiles and outliers. **(A)** women; **(B)** men.

Emotional valence ratings were conducted for the six basic emotions defined by Ekman (happiness, anger, disgust, fear, sadness, surprise; Ekman et al., 1972, Ekman and Friesen, 1975, 1978). The distribution of the emotional valence ratings across scenes is depicted in **Figure 4**, separated by gender. A MANOVA with the six emotions as independent variables and gender and scene as fixed factors suggested a small but significant tendency for men to rate the images somewhat higher with respect to all six emotions (main effect of gender: Wilk’s λ = 0.978, *F*_6,11205_ = 42.83, *p* < 0.001; interaction gender * scene: Wilk’s λ = 0.868, *F*_6,11205_ = 1.21; *p* < 0.001). However, *post hoc* univariate tests revealed that gender effect could not be observed for disgust (*F*_1,11210_ = 0.610, *p* = 0.435), but for all other emotions (all *F* > 14.20, all *p* < 0.001). Interaction effects reflecting gender differences in the rating of individual scenes were observed for anger, fear, and sadness (all *F* > 1.19, all *p* < 0.037), but not for happiness, disgust, and surprise (all *F* < 1.085, all *p* > 0.202). Detailed descriptive statistics of the emotional valence ratings (mean, median, mode, standard deviation, skewness, standard deviation of skewness, curtosis, standard deviation of curtosis) for each scene are available along with the stimulus set.

#### Cognitive and Affective ToM Ratings

**FIGURE 5:**
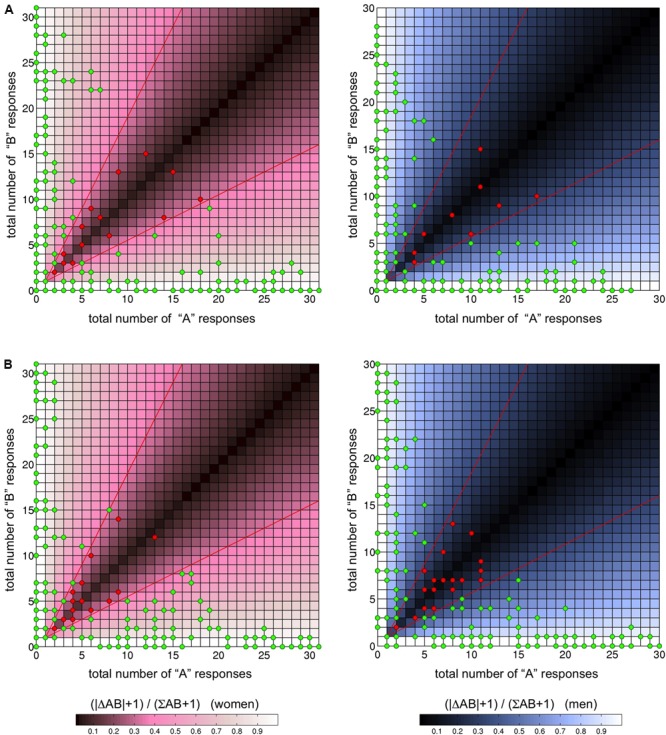
**Results for third-person cognitive (“Who can see more people?”, **A**)** and third-person affective (“Does person A or B feel better?”, **B**) condition, separated by gender. The shading reflects the function | ΔAB+1| /| ΣAB+1|, with the red line showing the value 1/3. The majority of the pictures yielded unambiguous responses (green dots), whereas the number of scenes rated as ambiguous ranged from 9 to 19.

To obtain a measure of ambiguity with respect to the ToM tasks (cognitive: “Can person A or person B see more people”; affective: “Does person A or B feel better”), we computed a simple measure of agreement, namely the ratio of the difference to the sum of A versus B responses (+1 to avoid division by 0: |ΔAB+1|/| ΣAB+1|). Scenes yielding values lower than 1/3 were considered ambiguous with respect to the participants’ responses. **Figure 5** displays the results of our evaluation, separated by the condition gender. In the cognitive ToM condition, 15 photographs came out as ambiguous among female participants, and nine among male participants. In the affective ToM condition, 19 images came out as ambiguous in both men and women, although there was only partial overlap. Supplementary Table S4 lists the potentially ambiguous scenes, separated by task and gender.

Note that the “both equally” responses were not considered in this approach, and users of the stimulus set may choose to include “ambiguous” scenes in an experiment when the “both equally” answer was the most common one in the group. Cumulative response data for each scene are available as along with the stimulus set.

#### Correlations of Stimulus Ratings and Psychometric Data

**FIGURE 6:**
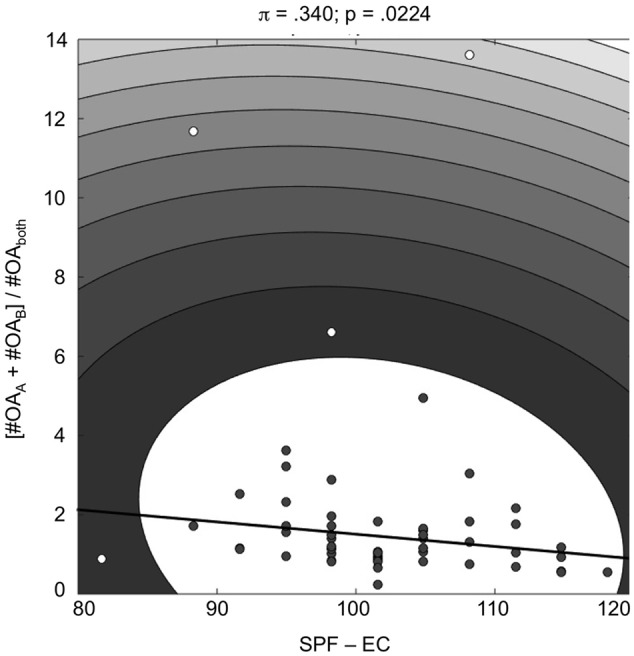
**Correlation of the SPF subscale *empathic concern* (SPF – EC) with decisiveness, i.e., the ratio of unambiguous responses (“person A” or “person B”) to ambiguous responses (“both”), in the other-affective condition ([OA_A_ + OA_B_]/OA_both_).** The plot depicts a robust Shepherd’s Pi correlation (Schwarzkopf et al., 2012).

To assess a potential relationship between response behavior during stimulus evaluation and psychometric measures of self-reported social cognitive abilities, we computed numeric measures that reflected individuals’ “typical” response behavior across the stimuli. Across the cohort of study participants (*N* = 59, due to missing SPF and AQ data from two participants), we observed a significant negative correlation between the *empathic concern* subscale of the SPF (SPF – EC) and the decisiveness measure in the third-person affective condition (i.e., the tendency to decide for either person A or B to feel better versus choosing the option “both equally”; Spearman’s *r* = -0.30375; *p* = 0.0193). This correlation remained significant when bivariate outliers were excluded by bootstrapping the Mahalanobis distance (*Shepherd’s Pi* correlation; Schwarzkopf et al., 2012; see **Figure 6**). No other correlations between stimulus ratings and psychometric data reached significance (all *p* > 0.30).

## Discussion

We have developed a photograph-based normative stimulus set (*The ToMenovela*) specifically designed for the experimental assessment of social cognition, particularly suitable for neuroimaging studies. All stimuli were designed in a way that (a) ecological validity would be high and (b) different types of ToM- and empathy-related constructs can be assessed experimentally (i.e., affective empathy, affective ToM (≈ cognitive empathy) and cognitive ToM; see Walter, 2012). The stimulus set will be available for non-commercial research free of charge for other researchers upon contacting the authors.^5^

### Applicability to the Study of Social Cognition

Our focus during the generation of the here presented stimulus set was high ecological validity. To this end, we scripted a background story and individual scenes revolving around a fictional circle of friends, the eight main characters. The scenes all depict at least two of the eight protagonists, but are yet independent of each other, showing the characters in different combinations and across a variety of different social situations and locations. While certain basic characteristics are fixed due to the nature of the stimulus set (e.g., the age of the protagonists in the twenties or early thirties, or the urban setting of the scenes), it should readily be possible for an experimenter to adapt the background story to their requirements.

By using a plausible real-life setting, our stimulus set bears some similarity with the MASC, a movie-based test instrument for the study of social cognition (Dziobek et al., 2006). While the MASC has previously constituted a considerable advance in ecological validity of test instruments of social cognitive processing, it is not without limitations. Its fixed composition as a movie of people at a dinner party limits the spectrum of emotions displayed and the use of non-social control tasks. These two limitations are less prominent in the MET (Dziobek et al., 2008) and in the cartoon-based ToM task developed by Schnell et al. (2011) and Walter et al. (2011), but the ecological validity of those tasks is on the other hand limited by the somewhat artificial construction of the MET stimuli and the lack of facial expressions in the cartoon-based task. Here, we provide a stimulus set that combines a plausible ecological setting with a broad range of emotions displayed across stimuli and the possibility to apply different tasks to the same stimuli.

One important limitation of the present stimulus set may be the ethnic background and age range of the eight main characters. First, the ethnic composition was rather narrow, albeit somewhat representative for a European urban area (seven Europeans, one East Indian), which may be an advantage when testing the typically available study population in Europe (or, to some extent, North America or Australia), namely, drawing from the student body of the researchers’ institution (Henrich et al., 2010), but may limit the interpretation when using the stimulus set with a non-Western study population (Adams et al., 2010; Koelkebeck et al., 2011; Hu et al., 2015). Similar considerations apply with respect to age. The protagonists of the *ToMenovela* are all in their twenties or early thirties. They may thus be highly comparable to the typical cohort of participants in psychological experiments at educational institutions (Henrich et al., 2010). As the biographies were written with considerations to our anticipated study populations, we cannot exclude that the biographies provided may have influenced the ratings. Future experimenters may further improve the comparability by adapting the characters’ biographies to their specific study populations, although it must be cautioned that doing so might warrant the collection of new normative data. The authors had considered the inclusion of elderly protagonists in the stimulus set, to make it more approachable by older study participants. That would, however, raise the potential confound that the (healthy) elderly are generally capable of imagining or retrieving information from memories of their own youth, while younger participants cannot to the same extent imagine themselves as being old. The authors are aware of the limitation that may arise when applying our stimulus set to a study population that differs substantially from our protagonists with respect to age, ethnicity, or cultural background. We strongly encourage researchers to expand our stimulus set presented here by including other ethnicities or age groups, paving the way for investigations of individual differences in social cognition.

With respect to the 8-SIF framework, it must be noted that the *ToMenovela*, does not contain any immediate (written or auditory) verbal information. Therefore, the factors I2 and I4 of the 8-SIF, the *immediate linguistic information* about agents or context, could not be implemented in our stimulus set, at least in its present form. While the authors do understand that this may constitute a potential limitation, it should be noted that all images were intended to be comprehensible without verbal information, and preliminary analyses of the free-text responses in our validation study confirm that the content of the images was indeed understood by the participants.^6^ We encourage future researchers interested in factors I2 and I4 of the 8-SIF to expand the stimuli by adding – spoken or written – verbal information to the photographs.

### Normative Evaluation

During our normative data collection, each scene was rated with respect to principal content, cognitive and affective ToM, and to first-person emotional salience and valence – the latter with respect to the six basic emotions according to Ekman (Ekman and Friesen, 1975). Ratings were performed by 61 participants (31 women, 30 men). Women and men in our sample were highly comparable with respect to age, education, intelligence, depressiveness, trait anxiety, anger, and impulsivity. In line with previous studies, autism-related traits were more pronounced in male participants scores, while men scored lower in several subscales of the empathy-related questionnaires (fantasy, empathic concern, personal distress, and sum scores, but not perspective taking). Supplementary Table S5 displays an overview of the tasks employed during evaluation and their potential applications in future research.

#### Emotional Salience and Valence

Analysis of the salience ratings (“How much do you feel affected by the picture?”) revealed a median rating of approximately 30 percent with a broad range from approximately 10 to 60 percent (**Figure 3**). The relatively low median arousal with a broad range was not unexpected, as the authors had aimed to depict real-life situations and interactions in the stimulus set. Along the same line, the rating of the scenes with respect to basic emotions revealed that happiness was most strongly represented across the stimuli, while, for example, only few scenes received high ratings for disgust (**Figure 4**). Importantly for future users of our stimulus set, all six emotions were represented in subsets of the scenes, and researchers can select the subset of pictures suitable for certain specific research questions.

We found small but significant gender difference of the ratings: men tended to rate the images somewhat higher with respect to emotional salience (*first*-*person affectiv*e: “How much do you feel affected by the picture?”) and to all emotion-ratings except for disgust. As shown in the *post hoc* univariate tests, gender differences could not be observed for disgust, but for all other emotions requested. Surprisingly, rather few studies have thus far investigated gender differences in emotion processing. One previous study using images from the IAPS (Lang et al., 1998) suggested that women had a higher tendency to rate pictures as fearful (Barke et al., 2012) or found no gender differences at all (Gruhn and Scheibe, 2008). With respect to happiness – and possibly surprise – ratings, on the other hand, our results are in line with previous studies that have shown men to rate pictures more positively (Barke et al., 2012), particularly pictures with erotic content (Bradley et al., 2001). Our stimulus set, while not displaying explicit nudity, does contain scenes with (in most cases implicit) erotic content that might have contributed to the overall more positive ratings by male participants. It must be cautioned, however, that the scenes were not designed to elicit extreme emotional responses as is the case with the IAPS pictures. Therefore, further research is required to systematically characterize the gender differences observed here. Finally, the authors would like to emphasize that all differences observed were, albeit being significant, quantitatively small and should therefore be unlikely to affect the usability of our stimulus set. Furthermore, we did not include experts like psychotherapists or people well versed in the *Facial Action Coding System* (FACS, Ekman and Friesen, 1978) to evaluate the pictures from a rather professional point of view and thereby we do not deliver a gold-standard for salience and valence norms.

#### Results on Third-Person ToM: Agreement across Raters

Analysis for the cognitive and affective ToM conditions revealed that only a small subset of images yielded ambiguous responses. In the cognitive condition (“Who can see more people?”), 15 photographs were rated as ambiguous among female participants, and nine among male participants (Supplementary Table S4). In the affective ToM condition (“Does person A or B feel better?”), nineteen images were rated as ambiguous by both men and women, although there was only partial overlap. Depending on future researchers’ need for unambiguous stimulus material, scenes with little or no disagreement can be selected from our stimulus set. The detailed results of the rating procedure are available along with the stimulus set. It should be noted at this point that a certain degree of ambiguity of the scenes may be unavoidable, given that our focus was on ecological validity of the stimulus material, and ambiguity of certain stimuli is most likely not unique to the *ToMenovela*. For example, rating studies of the well-established IAPS stimuli suggest that several pictures did not receive high ratings on the initially intended emotions in a normative rating procedure (Barke et al., 2012). On the other hand, some researchers may want to explicitly include ambiguous scenes, for example in order to vary cognitive load or task difficulty. Most ToM or mentalizing tasks currently used simplified settings, unimodal structures or highly simplified fictional characters. As mentalizing can be conceptualized as “an executive component managing the multiple aspects of representations that are concurrently activated by the inherently complex everyday social interactions” (Brunet-Gouet et al., 2011), we suggest that the naturalistic setting employed in our paradigm invariably includes some degree of ambiguity, at least in a subset of the stimuli, while rather accurately representing daily life social interactions.

#### Relationship of Stimulus Ratings with Self-report Measures of Social Cognition

Correlational analyses revealed a negative relationship between decisiveness in the third-person affective condition and the empathic concern subscale of the SPF (**Figure 6**). This may appear somewhat surprising, as this negative correlation suggests that participants with higher empathic concern show more difficulties in judging an individual’s emotion. On the other hand, there is considerable debate with respect to potential subdivisions of the ToM construct into different subprocesses like emotion recognition, understanding of causality, or the ability to distinguish knowledge and facts (Kanske et al., 2015). Furthermore, a distinction has been suggested between affective empathy, affective ToM/cognitive empathy, and cognitive ToM (Walter, 2012; Schaafsma et al., 2015). Kanske et al. (2015) could recently demonstrate that empathy and ToM can be orthogonalized within the same task at both the behavioral and neural level. With respect to the present results, this notion points to the possibility that increased empathic concern may induce difficulties in some individuals when it comes to making (comparative) decisions about other people’s feelings. One limitation in this context is that we did not record reaction time data, which would provide a more objective measure to further substantiate this interpretation.

### Limitations and Directions for Future Research

It should be noted that, as of now, expert evaluation of the *ToMenovela* has not been completed, and thus the stimulus set does not represent a performance test as of yet, which can be used for investigating mentalizing skills or deficits at the behavioral level. Future studies are planned that will obtain both expert ratings on the stimulus set and ratings from clinical populations like individuals with autism spectrum disorders, both of which will be used to establish concurrent and discriminant validity. In addition, other researchers may develop new questions applicable to our stimulus set, for example with respect to social cue recognition or potential gender-related differences in ToM for male versus female characters. We have summarized the purpose of each question used in the initial evaluation, along with potential use cases in Supplementary Table S5, in order to provide suggestions for future applications of the *ToMenovela* stimuli.

### Availability

The *ToMenovela* stimulus set is freely available for use in non-commercial scientific research. Functionalities of this online service include the picture set in three different resolutions, full normative data and the full quiz. To prevent circulation of the pictures unrelated to research usage, scientists will be requested to provide contact details and a brief outline of their research purpose when accessing to the *ToMenovela* database. All details required for access can be found at http://neuro2.med.uni-magdeburg.de/~bschott/ToMenovela. The script of the scenes is available in German language only and can be obtained from the first author (maike.herbort@charite.de).

## Ethic Statement

The study was approved by the Ethics Committee of the Otto von Guericke University, Magdeburg, Faculty of Medicine. All actors gave written informed consent for the use of the resulting photographs for research purposes. All participants of the evaluation study gave written informed consent prior to the participation in the study in accordance with the Declaration of Helsinki. Some photographs display children as supporting actors. All parents were informed about the purpose of the stimulus set and consented to have their children participate in the photo shootings. At least one parent or (in case of children over 10), a person entrusted by the parents, was always present when photographs involving children were taken. No children served as supporting actors in photographs with potentially disturbing content (e.g., accidents, fighting, sexually suggestive scenes).

## Author Contributions

MCH, BR, HW, and BHS designed research; MCH, BR, JI, CS, and NG performed research; CS programmed the stimulus rating software; MCH, JI, CS, TW, and BHS analyzed the data; RH and ID supervised evaluation of stimulus material and data analysis; MCH, HW, ID, and BHS wrote the paper. All authors approved the final version of the manuscript.

## Conflict of Interest Statement

The authors declare that the research was conducted in the absence of any commercial or financial relationships that could be construed as a potential conflict of interest.

## Footnotes

1 http://www.cogsci.nl/stimulus-sets

2 http://www.lensbreaker.com

3 http://www.gimp.org

4 The original normative data of the SPF can be found at http://psydok.psycharchives.de/jspui/handle/20.500.11780/3343

5 Please contact us via the ToMenovela website (http://neuro2.med.uni-magdeburg.de/∼bschott/ToMenovela) to gain access to the stimulus set.

6 Please note that one picture (#164), for which the free-text responses suggested ambiguity of content, was excluded from the stimulus set for that reason.

## Supplementary Material

FIGURE S1 **Example trial of the normative data collection study**.
