# Supplementary material for: The ToMenovela – A Photograph-Based Stimulus Set for the Study of Social Cognition with High Ecological Validity
Source: Front Psychol. 2016 Dec 2;7:1883. doi: 10.3389/fpsyg.2016.01883 (PMC5133259; doi:10.3389/fpsyg.2016.01883)
Supplement: FIGURE S1 — Example trial of the normative data collection study. [file Image_1.PDF]

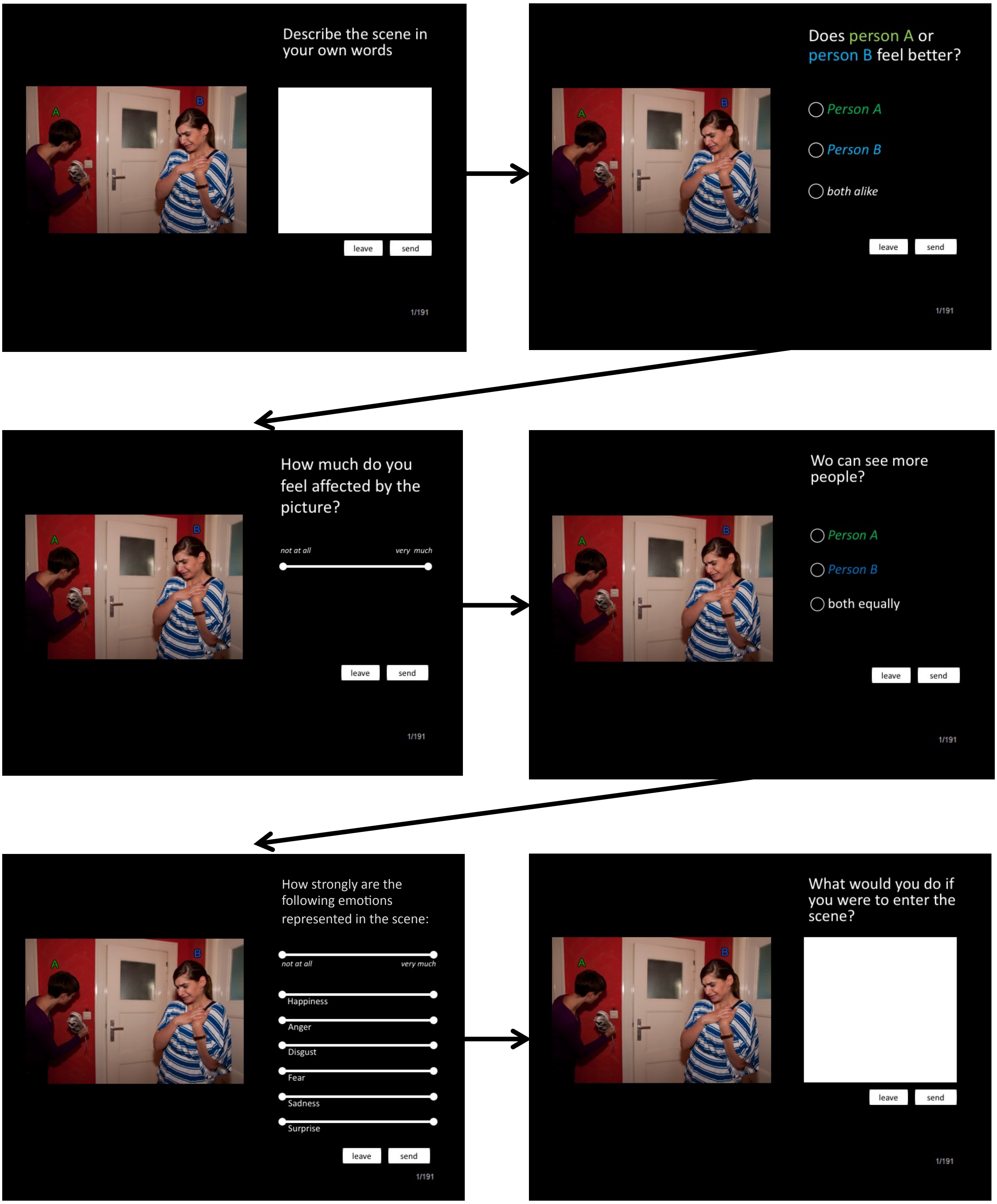

Describe the scene in your own words
A
B
leave
send
1/191
Does person A or person B feel better?
Person A
Person B
both alike
leave
send
1/191
How much do you feel affected by the picture?
not at all
very much
leave
send
1/191
Wo can see more people?
Person A
Person B
both equally
leave
send
1/191
How strongly are the following emotions represented in the scene:
not at all
very much
Happiness
Anger
Disgust
Fear
Sadness
Surprise
leave
send
1/191
What would you do if you were to enter the scene?
leave
send
1/191
