# Supplementary material for: The ToMenovela – A Photograph-Based Stimulus Set for the Study of Social Cognition with High Ecological Validity
Source: Front Psychol. 2016 Dec 2;7:1883. doi: 10.3389/fpsyg.2016.01883 (PMC5133259; doi:10.3389/fpsyg.2016.01883)
Supplement: Supplementary file 2 [file Data_Sheet_1.PDF]

Supplementary Table S1A: Example scene 1.

**Dinner with friends**	
Content	Joy! Pleasure! Cool time
Emotion 1 (Perspective 1)	Amusement
Emotion 2 (Perspective 2)	Joy
Emotion 3 (Perspective 3)	Companionship
Setting	Big table with happy people (other possibility: image of a private get-together); hosts are handing over food and drinks, maybe someone enters the room with a big cooking pot, another one serves wine; plates are already filled with salad
Actors	All 8 main characters
Requisites	Dishes, silverware<br>Spaghetti<br>Sauce<br>Salad, salad bowl, salad servers<br>Water glasses<br>Wine glasses, wine bottles<br>2 beer bottles
Location	Flexible, most likely at Oliver's and Theresa's place
Mindset actor 1	Haven't had such a great party for a while ...how cool that everybody is here! It's a real good party, everybody seems to enjoy it and have fun. I should serve some more wine anyway.
Mindset actor 2	My stay overseas was so amazing, and finally I can tell my friends about it! → lost in conversation with 2 others
Mindset actor 3	Oh my, this guy has been talking about his stay for hours now, doesn't he get that he repeats himself? Well ... come on, be a nice buddy and keep listening
Mindset actor 4	Hooray! There's nothing better than hanging out with the guys and making silly jokes. My colleagues are just laughing at *me* when I'm doing this old chestnut, but my man here is gettin' it just right! → doing voluminous gestures in an exposed position
Mindset actor 5 and more	Laughing about jokes of actor 4: he really has the best kind of humor / so funny the way he can tell his stories → sitting in a semicircle around him

The table displays a scene translated from the original German script.

Supplementary Table S1B: Example scene 2.

**Funeral**	
Content	Funeral
Emotion 1 (Perspective 1)	Sadness
Emotion 2 (Perspective 2)	Anger
Emotion 3 (Perspective 3)	Emptiness
Setting	Cemetery
Actors	Woman2, Man3, background actors (male+2 female), reverend
Requisites	Morning garments, hat, rather elegant, sunglasses (black!)
Location	Graveyard near Hasenheide (Berlin-Kreuzberg)
Note	Make sure that no one looks "lost"; be aware to have a smart angle concerning the anticipated coffin
Mindset Woman2	How can we just go on? Nothing makes sense without her. I will miss her so much, I can like literally hear her laughing and cheering whenever I told her a good joke → tears
Mindset Man3	Well, actually I didn't really know her…but one of us had to go with [Woman2]. Hope it's over soon, and afterwards there be something delicious to eat…And hopefully I look really concerned right now…
Mindset actor 3	Oh, if this stupid idiot of an ambulance driver had just arrived 2 minutes earlier! Maybe she would still be alive! Maybe she could have been saved! →furious facial expression
Mindset actor 4	What is this all good for? It will not bring her back! I'd rather be home in my warm bed right now, watching some relaxing DVD or something… What did the reverend just say?

The table displays a scene translated from the original German script.

Supplementary Table S2: Example questions from the quiz.

Example question	Answer options
Who could you ask for styling and outfit advice?	A: Catherine<br>B: Celine
The guys like to meet …	A: for an after-work beer<br>B: to watch wrestling together
Who would join in for an adventure trip immediately?	A: Celine<br>B: Leah
Who has just completed law school?	A: Oliver<br>B: Noah

Seven days (+/- 2 days) prior to the experiment, participants was sent the biographies and relation chart. In order to ensure familiarity with the circle of friends, participants were asked to complete a paper-pencil test (44 two-alternative forced-choice questions) on the day of the experiment. Overall, 2 participants were excluded after failing the test.

Table S3: Detailed characteristics of the scenes.

Pic_ID	Scene Name (German)	Scene Name (English)	M1	M2	M3	M4	F1	F2	F3	F4	BM	BF	BC	in	out
**001**	3:30 aufstehen	Rise and Shine	1	0	0	0	1	0	0	0	0	0	0	1	0
**002**	Frisch verliebt	Newly in love	0	0	0	1	0	0	0	1	0	0	0	0	1
**003**	Achtung, Chef im Anmarsch!	Watch out, boss is comin'	0	0	0	0	0	1	1	0	1	0	0	1	0
**004**	Adventskranz	Advent wreath	0	0	0	0	0	1	1	0	0	0	0	1	0
**005**	Am Ticketautomaten	Ticket vending machine	0	0	1	1	0	0	0	0	0	0	0	1	0
**006**	Anstoßen	Congrats	0	1	1	0	0	0	0	0	0	0	0	1	0
**007**	Apotheke	At the pharmacy	0	0	0	0	1	0	1	0	0	0	0	1	0
**008**	Archivarbeit	Archiving	1	0	0	0	1	0	0	0	0	0	0	1	0
**009**	Armdrücken	Arm wrestling	0	0	1	1	0	0	0	1	0	0	0	0	1
**010**	Morgens im Bad	Morning routine	0	0	0	0	0	1	1	0	0	0	0	1	0
**011**	Auf dem Markt	At the market	1	0	0	0	1	0	0	0	0	0	0	0	1
**012**	Auf der Rolltreppe	Escalator	0	1	1	0	0	0	0	0	0	0	0	0	1
**013**	Auf geht's zum Zelten	Let's go camping	0	0	0	0	0	1	1	0	0	0	0	0	1
**014**	Ausheulen	A shoulder to cry on	0	1	0	0	1	0	0	0	0	0	0	0	1
**015**	Auto kommt angefahren – STOP!	Watch out! Car!	1	0	0	0	1	0	0	0	0	0	0	0	1
**016**	Fahrradunfall	Bicycle accident	1	0	0	0	1	0	0	0	0	1	0	0	1
**017**	Baby it's cold outside	Baby it's cold outside	0	0	0	0	0	1	1	0	0	0	0	1	0
**018**	Babygeschenk	Baby's first present	0	0	0	0	1	0	0	1	0	0	0	1	0
**019**	Babysitting	Babysitting	1	0	0	0	1	0	0	0	0	0	1	1	0
**020**	Barbecue auf Balkon	BBQ	1	0	1	1	0	0	0	0	0	0	0	0	1
**021**	Beerdigung	Funeral	0	1	1	0	1	0	1	0	0	1	0	0	1
**022**	Bei Oma	Grumpy granny	0	0	0	1	0	0	0	1	1	1	1	1	0
**023**	Beim Rätsel lösen	The riddle	1	1	0	0	0	0	0	0	0	0	0	0	1
**024**	Beim Schnellimbiss	Fast food	0	0	1	1	0	0	0	0	0	0	0	0	1
**025**	Bewerbungsfotos	Application photo	1	0	0	0	1	0	0	0	0	0	0	1	0
**026**	Billard	Playing pool	0	0	1	1	0	0	1	0	0	0	0	1	0

**027**	Blei gießen	Lead-pouring	1	1	1	1	0	1	0	1	0	0	0	1	0
**028**	Blumen pflanzen	Planting flowers	1	0	0	0	1	0	0	0	0	0	0	0	1
**029**	Ich hab dich gern	Love actually	0	0	0	1	0	0	0	1	0	0	0	0	1
**030**	Blut abnehmen	Blood drawing	1	0	0	0	1	0	0	0	1	0	0	1	0
**031**	Brief einwerfen	Posting a letter	0	1	1	0	0	0	0	0	0	0	0	0	1
**032**	Businessmeeting	Business meeting	0	0	1	0	0	0	1	0	0	0	0	0	1
**033**	Bye bye Döner	Bye Bye lunch	1	1	1	0	0	0	0	0	0	0	0	0	1
**034**	Cheers	Cheers	0	1	1	0	0	1	1	0	0	0	0	1	0
**035**	Wütender Chef	Angry Boss	0	0	0	0	0	1	1	0	1	0	0	1	0
**036**	Cocktails mixen	Cocktails	0	1	1	1	0	0	0	0	1	0	0	1	0
**037**	Danke für die Blumen	Thanks for the flowers	0	0	0	1	0	0	0	1	0	0	0	1	0
**038**	Freundinnen beim Nähen	Sewing	0	0	0	0	1	0	1	0	0	0	0	1	0
**039**	Bild und Stimmung hängen schief	The picture is crooked	0	0	0	1	0	0	0	1	0	0	0	1	0
**040**	Dem Himmel so nah	Close to heaven	0	0	0	1	0	0	0	1	0	0	0	0	1
**041**	Der Absagebrief	Letter of refusal	1	0	0	0	1	0	0	0	0	0	0	1	0
**042**	Der Bankautomat	Cash point	0	1	1	1	0	0	0	0	0	0	0	0	1
**043**	Der Künstler vor seiner Leinwand	Touched by the muse	0	1	0	0	0	1	0	0	0	0	0	1	0
**044**	Der Kuss	Kiss me	0	0	0	1	0	0	0	1	0	0	0	0	1
**045**	Der Vortrag	The presentation	1	0	1	0	1	0	0	0	0	0	0	1	0
**046**	Der erste Streit	Trouble's brewing	0	0	0	1	0	0	0	1	0	0	0	1	0
**047**	Die Maske	The mask	0	0	1	0	0	0	0	1	0	0	0	1	0
**048**	Die Welt im Inneren bereisen	Envisioning the world	0	1	1	0	0	0	0	0	0	0	0	1	0
**049**	Eifersucht	Jealousy	0	0	0	1	0	0	0	1	1	0	0	0	1
**050**	Ein Brief von…ihm!	A letter from…him!	0	0	0	0	0	1	1	0	0	0	0	1	0
**051**	Ein Paar streitet sich	The quarrel	1	0	0	0	1	0	0	0	0	0	0	1	0
**052**	Ein romantisches Picknick	Romantic picnic	1	0	0	0	1	0	0	0	0	0	0	0	1
**053**	Eincremen	Skin care	0	0	0	0	0	1	1	0	0	0	0	1	0
**054**	Einkaufstüten schleppen	Heavy shopping bags	0	1	1	0	0	0	0	0	0	0	0	0	1
**055**	Ekel an der Wursttheke	Disgust at the meat counter	0	1	1	0	0	0	0	0	0	0	0	1	0

**056**	Frische Erdbeeren kaufen	Buying fresh strawberries	1	0	0	0	1	0	0	0	0	0	0	0	1
**057**	Liebesbrief verbrennen	Burning a love letter	0	0	0	0	0	1	1	0	0	0	0	0	1
**058**	Fahrrad kaputt	The flat tyre	0	0	0	1	0	0	0	1	0	0	0	0	1
**059**	Gefangen im Fahrstuhl	Stuck in the elevator	0	0	1	0	0	1	1	0	0	0	0	1	0
**060**	Familienfoto	Family portrait	0	0	0	1	0	0	0	1	2	3	1	0	1
**061**	Fenster putzen	Cleaning session	0	0	0	0	0	1	1	0	0	0	0	1	0
**062**	Planespotting	Plane-spotting	1	0	0	0	1	0	0	0	0	0	0	0	1
**063**	Wegbeschreibung	Directions	0	1	1	0	0	0	0	0	0	1	0	0	1
**064**	Frisbee im Park	Frisbee	0	1	0	0	0	0	1	0	0	0	0	0	1
**065**	Friseur	Hairdresser	0	0	0	0	0	1	0	1	0	0	0	1	0
**066**	Fußballfans	Go, Titans, go	0	0	1	1	0	0	0	0	2	0	0	1	0
**067**	Gassi gehen	Walk the dog	1	0	0	0	1	0	0	0	0	0	0	0	1
**068**	Geeks @ work	Geeks @ work	0	1	1	0	0	0	0	0	1	0	0	1	0
**069**	Geldbeutel verloren	Lost, not found	0	1	0	0	1	0	0	0	0	0	0	0	1
**070**	Gesunde Ernährung	Healthy diet	0	0	1	0	0	1	0	0	0	0	0	0	1
**071**	Glas nicht aufkriegen	Can't open that jar	1	0	0	0	1	0	0	0	0	0	0	1	0
**072**	Handyanruf – ewig drauf gewartet	The long-expected call	0	1	0	0	0	1	1	0	0	0	0	0	1
**073**	Die Kakerlake	The cockroach	0	1	1	0	0	0	0	0	0	0	0	1	0
**074**	Höhenangst	Vertigo	0	0	0	0	0	1	1	0	0	0	0	0	1
**075**	Hose offen	My pants!	0	0	1	1	0	0	0	0	0	0	0	0	1
**076**	Huete ausprobieren	New hats	0	0	0	0	0	1	1	0	0	0	0	0	1
**077**	Geburtstagskind	Birthday child	0	0	0	1	0	0	0	1	2	2	1	1	0
**078**	I love you	I love you	1	0	0	0	1	0	0	0	0	0	0	0	1
**079**	Koffer packen	Packing suitcases	1	0	0	0	1	0	0	1	0	0	0	1	0
**080**	Im Bücher-Antiquariat	At the book shop	0	1	1	0	0	1	0	0	0	0	0	1	0
**081**	Im Handyshop	The cell phone shop	1	0	1	0	0	0	0	0	0	0	0	0	1
**082**	Im Teegeschäft	The tea shop	0	1	0	0	0	1	0	0	1	0	0	1	0
**083**	Im Wartezimmer	Doctor's waiting room	1	0	0	0	1	0	0	0	0	0	0	1	0
**084**	In den Finger schneiden	Ouch! Cut my finger!	0	1	0	0	0	0	0	1	0	0	0	1	0

**085**	Der Hundehaufen	Dog poop	1	0	0	0	1	0	0	0	0	0	0	0	1
**086**	Ist das Kunst oder kann das weg?	That's probably...art	0	1	0	0	0	1	0	0	0	0	0	0	1
**087**	Hochzeitskleid aussuchen	The wedding dress	0	0	0	0	0	1	1	1	0	1	0	1	0
**088**	Langeweile	Boredom	0	0	1	0	1	0	0	0	0	0	0	0	1
**089**	Joggen gehen	Running	0	0	0	0	0	1	1	0	0	0	0	0	1
**090**	Jungs beim Zocken 1	Boys playing – pt I	1	1	0	1	0	0	0	0	0	0	0	1	0
**091**	Jungs beim Zocken 2	Boys playing – pt II	1	0	1	0	0	0	0	0	1	0	0	1	0
**092**	Kaffee trinken bei Oma	Tea Time with Granny	0	0	0	1	0	0	0	1	1	3	1	1	0
**093**	Kerze anzünden auf dem Friedhof	Candle of remembrance	0	1	0	0	0	0	1	0	0	0	0	0	1
**094**	Sportsfreunde	Sport buddies	1	0	0	1	0	0	0	0	1	0	0	0	1
**095**	Kicker	Foosball	1	0	1	1	0	0	0	0	1	0	0	1	0
**096**	Kirmes 1: Zuckerwatte	Fun Fair I - Sweet sweets	1	0	1	0	1	0	0	0	0	0	0	0	1
**097**	Kirmes 2: Autoscooter	Fun Fair II - Bumper Car	0	0	1	0	0	0	0	1	1	0	0	0	1
**098**	Kirmes 3: Übel fühlen	Fun Fair III - Sick	0	1	0	0	0	0	1	0	0	0	0	0	1
**099**	Kirmes 4: Herz schießen	Fun Fair IV - Heart booth	1	0	0	0	1	0	0	0	0	0	0	0	1
**100**	Kitzeln	Are you ticklish?	0	0	0	1	0	0	0	1	0	0	0	0	1
**101**	Klavier spielen	Piano – playing	0	1	0	0	1	0	0	1	0	0	0	1	0
**102**	Klavier spielen lernen	Piano – teaching	0	1	0	0	1	0	0	0	0	0	0	1	0
**103**	Kondom fällt aus Portemonnaie	Ooops, that was a condom	0	0	0	0	0	1	1	0	0	1	0	0	1
**104**	Kotzen ins Klo	Throwing up	0	1	1	0	0	0	0	0	0	0	0	1	0
**105**	Krankenbesuch	Visiting a sick friend	0	0	1	1	0	0	0	1	1	0	0	1	0
**106**	Krimi gucken	Crime time	0	0	0	0	1	1	1	1	0	0	0	1	0
**107**	Kuscheln mit Katze	Snuggle the cat	1	0	0	0	1	0	0	0	0	0	0	1	0
**108**	Sei nicht nervös	Don’t be stressed	0	1	0	0	1	0	0	0	0	0	0	1	0
**109**	Laufmasche in der Strumpfhose	A hole in her tights	0	0	0	0	0	1	1	0	0	0	0	0	1
**110**	Lesen, welch eine Lust!	Delightful reading	0	0	0	0	0	1	1	0	0	0	0	1	0
**111**	Liebe kennt kein Alter	Love knows no age	1	0	0	1	0	0	0	0	0	0	2	0	1
**112**	LP vom Flohmarkt	LP from the flea market	0	1	1	0	0	0	0	0	0	0	0	0	1
**113**	Mach den Mist doch alleine	DIY – if you can	0	0	0	1	0	0	0	1	0	0	0	1	0

**114**	Massage	Massage	0	0	0	1	0	0	0	1	0	0	0	0	1
**115**	Meditation	Meditation	0	0	0	0	1	1	1	0	0	0	0	1	0
**116**	Möbel aufbauen	Assembling furniture	0	0	0	0	0	1	1	0	1	0	0	1	0
**117**	Motorschaden	Engine breakdown	0	0	0	1	0	0	0	1	0	0	0	0	1
**118**	My head's gonna burst	My head's gonna burst	0	0	0	1	0	0	0	1	0	0	0	1	0
**119**	Nachhilfe geben	The tutor	0	1	1	0	0	0	0	0	0	0	1	1	0
**120**	Nachtschicht	Night shift	0	0	1	0	0	0	1	0	0	0	0	1	0
**121**	Nägel lackieren	Manicure	0	0	0	0	0	1	0	1	0	0	0	0	1
**122**	Oh…äh…danke…	Um...er...thanks	1	0	0	0	1	0	0	0	0	0	0	1	0
**123**	Heißer Typ	The hottie	0	0	0	0	0	1	1	1	1	0	0	0	1
**124**	Oh...ein Rohkostteller...lecker	Raw food is healthy	0	1	0	1	0	0	0	1	0	0	0	1	0
**125**	Oldtimer fahren	Vintage car	0	0	0	1	0	0	0	1	0	0	0	0	1
**126**	Paket	The parcel	0	1	1	0	0	0	0	0	0	0	0	1	0
**127**	Poker	Poker	0	0	1	1	0	0	0	1	1	0	0	1	0
**128**	Sexy Sekretärin	The sexy secretary	0	0	1	1	0	0	0	0	0	1	0	1	0
**129**	Portrait einer Schwangeren	Portrait of a mum-to-be	1	0	0	0	1	0	0	0	0	0	0	0	1
**130**	Puzzeln	Puzzling	1	0	0	0	1	0	0	0	0	0	0	1	0
**131**	Renovieren mit Flirt	Refurbish with benefit	0	0	0	1	0	0	0	1	0	0	0	1	0
**132**	Samba!	Samba!	0	0	0	1	0	0	0	1	0	0	0	1	0
**133**	Fernsehabend mit der Familie	TV family evening	0	0	0	1	0	0	0	1	1	1	1	1	0
**134**	Schach spielen	Chess	1	1	0	0	0	0	0	0	0	0	0	1	0
**135**	Schatz, du hast gebacken?!	Yummy cake, darling!	1	0	0	0	1	0	0	0	0	0	0	1	0
**136**	Schatz, hab ich zugenommen?	Gain or loss	0	0	0	1	0	0	0	1	0	0	0	1	0
**137**	Findest du das wirklich gut?	Do you really like that?	0	0	0	1	0	0	0	1	0	0	0	0	1
**138**	Scherben bringen Glück	Break a thing – mend your luck	0	0	0	1	0	0	0	1	0	0	0	1	0
**139**	Schlafend in der U-Bahn	Sleeping beauty	1	1	1	0	0	0	0	0	0	0	0	1	0
**140**	Schlechte Nachrichten	Bad news	0	0	0	1	0	0	0	1	1	0	0	1	0
**141**	Schlüssel vergessen	Forgot my keys	0	0	0	1	0	0	0	1	0	0	0	1	0
**142**	Schneeballschlacht	Snowball fight	0	0	1	1	0	0	0	1	0	0	0	0	1

**143**	Schnupfen, Husten, Heiserkeit	Caught a cold	0	0	0	1	0	0	0	1	0	0	0	1	0
**144**	Schwangere nerven	Annoying mum-to-be	0	0	0	0	1	0	1	0	0	0	0	0	1
**145**	Wellness zu Hause	Wellness	0	0	0	0	0	1	1	0	0	0	0	1	0
**146**	Sex	Sex	1	0	0	0	1	0	0	0	0	0	0	1	0
**147**	Sexy Outfit	Sexy outfit	0	0	0	0	0	1	0	1	1	0	0	0	1
**148**	Siegertreppchen	The winner takes it all	0	0	0	0	0	1	1	0	0	1	0	0	1
**149**	Überraschung!	Surprise!	0	0	0	1	0	0	0	1	0	0	0	1	0
**150**	Sinnierend	Thinking	1	1	0	0	0	0	0	0	0	0	0	0	1
**151**	Song schreiben	Songwriting	0	1	0	0	0	1	0	0	0	0	0	0	1
**152**	Sonniger Sommer	Sunny summer	0	0	0	0	1	1	1	1	0	0	0	0	1
**153**	Spaghettiessen mit Freunden	Dinner with friends	1	1	0	0	1	0	1	1	0	0	0	1	0
**154**	Spieleabend	Playing a game	0	0	0	0	1	0	1	1	0	1	1	1	0
**155**	Spielplatz	At the playground	0	0	0	1	0	0	0	1	0	0	0	0	1
**156**	Sport im Park	Exercising in the park	0	0	1	0	0	0	1	0	0	0	0	0	1
**157**	Spülen & Abtrocknen	Washing dishes	0	0	0	0	1	1	0	0	0	0	0	1	0
**158**	Stau	Traffic jam	0	0	0	0	0	1	1	0	0	0	0	0	1
**159**	Strandnixen	Summer beauties	0	0	0	0	0	1	1	1	0	0	0	0	1
**160**	Streit	The fight	1	0	1	0	0	0	0	0	0	0	0	0	1
**161**	Tag im Zoo	At the zoo	1	0	0	0	1	0	0	0	1	1	1	0	1
**162**	Tankstelle	Filling station	0	0	1	1	0	0	0	0	0	0	0	0	1
**163**	Tee-Zeremonie	Tea time	0	1	0	0	0	1	0	0	0	0	0	1	0
**165**	Träume im Möbelhaus	A furniture dream	0	0	0	1	1	0	0	1	0	0	0	1	0
**166**	Treppen hat der Teufel gemacht	Satan made these stairs	0	0	1	1	0	0	0	1	0	0	0	1	0
**167**	Kostümparty	Costume party	0	1	0	1	1	1	0	0	0	1	0	1	0
**168**	Trösten	Comforting a friend	0	0	0	0	0	1	1	0	0	0	0	1	0
**169**	Twister	Let's do the twister	0	1	1	0	0	1	0	1	0	0	0	0	1
**170**	Überraschungsei	Kinder surprise	1	0	1	0	0	0	0	0	0	0	0	1	0
**171**	Gefährliche High Heels	Dangerous high heels	0	0	1	1	0	0	0	1	0	1	0	0	1
**172**	In der Badewanne	Having a bath	1	0	0	0	1	0	0	0	0	0	0	1	0

**173**	Verängstigt im Kino	Scary movie	0	0	0	1	0	0	0	1	0	0	0	1	0
**174**	Vergorene Milch	Rotten milk	0	1	1	0	0	0	0	0	1	0	0	1	0
**175**	Liebespaar auf Reisen	Travelling lovers	0	0	0	1	0	0	0	1	0	0	0	1	0
**176**	Vertragsunterzeichnung	Signing the contract	1	0	0	0	1	0	0	0	1	0	0	1	0
**177**	Wäsche aufhängen	Hanging out the laundry	1	0	0	0	1	0	0	0	0	0	0	0	1
**178**	Wassermelone essen	Watermelons	1	1	0	0	1	0	0	0	0	0	0	0	1
**179**	Wasserpfeife für zwei	Shisha for two	1	0	0	1	0	0	0	0	0	0	0	0	1
**180**	Wechselgeld	Keep the change	0	1	0	0	0	0	1	0	0	0	0	1	0
**181**	Weinend auf einer Bank	Crying	0	1	1	0	0	0	0	0	0	1	0	0	1
**182**	Schönheit braucht Zeit	Beauty takes time	0	0	0	1	0	0	0	1	0	0	0	1	0
**183**	Weihnachtszeit	Christmas time...	0	0	0	0	0	1	1	1	0	0	0	1	0
**184**	Wo ist meine Brille?	Where are my glasses?	0	1	1	0	0	0	0	0	0	0	0	1	0
**185**	Dealer im Park	Drug dealers	1	0	0	0	1	0	0	0	2	0	0	0	1
**186**	Wir sind schwanger!	Positive pregnancy test	1	0	0	0	1	0	0	0	0	0	0	1	0
**187**	Wohnungsanzeigen	Apartment listings	0	1	1	0	0	0	0	0	0	0	0	0	1
**188**	Zahnarzt	Dentist	0	1	0	0	1	0	0	0	0	0	0	1	0
**189**	Zeit für ein Bad	Time for a dip	0	0	0	1	0	0	0	1	0	0	0	0	1
**190**	Zug verpasst	Missed the train	0	0	0	0	1	0	0	1	0	0	0	0	1
**191**	Zum ersten Mal Sushi	Sushi first-timer	0	0	0	1	0	0	0	1	0	0	0	0	1

Pic_ID = picture number; *M1* = Man 1 = Oliver; *M2* = Man 2 = Jonas / Noah; *M3* = Man 3 = Viktor / Victor; *M4* = Man 4 = Hannes / Jack
F1 = Woman 1 = Theresa; *F2* = Woman 2 = Kathrin / Catherine; *F3* = Woman 3 = Lea / Leah; *F4* = Woman 4 = Celine;
BM = number of background actors (male); *BF* = number of background actresses (female); *BC* = number of background actors / actresses (children);
in = indoor scene; *out* = outdoor scene

Supplementary Table S4: Ambiguous scenes.

	Other Affective	Other Cognitive	Intersection
Male	#: 19	#: 9	
	ID: 13, 20, 23, 36, 50, 59, 60, 70, 82, 85, 101, 116, 126, 134, 138, 153, 181, 183, 185	ID: 16, 24, 26, 95, 96, 99, 127, 159, 189	
Female	#: 19	#: 15	#: 1
	ID: 20, 22, 24, 28, 63, 81, 82, 90, 101, 105, 115, 119, 122, 153, 161, 174, 180, 183, 185	ID: 9, 16, 34, 36, 63, 87, 96, 124, 127, 142, 143, 144, 152, 158, 165	ID: 63
Intersection	#: 6	#: 3	
	ID: 20, 82, 101, 153, 183, 185	ID: 16, 96, 127	

For each gender and condition respectively, an index of response ambiguity was computed. Based on a simple calculation ($|\Delta AB+1|/|\Sigma AB+1|$), scenes with values lower than $^{1}/_{3}$ were considered ambiguous. All ambiguous scenes are listed here.

Intersection = common pictures of the respective column or row; *#* = number of pictures, *ID* = picture number

Supplementary Table S5: Overview of tasks employed during normative evaluation and potential applications

Task	Answer format	Operationalization of condition	Appropriate for quantitative assessment of…	Use Case
A) Describe the scene in your own words.	Open answer format	General understanding<br>Memory<br><br>Social understanding	• Comprehension of social interactions<br>• Memory (e.g. level of details remembered)	• identifying social comprehension<br>• testing memory for social situations
B) Does person A or person B feel better?	Multiple Choice<br>o Person A<br>o Person B<br>o both alike	Affective ToM (3rd person perspective)<br><br>Emotion Recognition	• physiological correlates (normative data can help picking pictures that are not/are ambiguous)	• identifying affective ToM network in healthy controls (contrasting this condition with a HLB condition, e.g. showing the same pictures asking for a gender judgment) using fMRI
C) Who can see more people?	Multiple Choice<br>o Person A<br>o Person B<br>o both equally	Cognitive ToM (3rd person perspective)<br><br>Visual Perspective Taking	• physiological correlates (normative data can help picking pictures that are not/are ambiguous)	• comparing brain activation for cognitive ToM between individuals with ASD and controls
D) How much do you feel affected by the picture?	Visual analog scale, designed as a slider, ranging from "not at all" to "very much"	Emotional reactivity (1st person perspective)<br><br>Affective Empathy	• behavior: individual differences in emotional reactivity<br>• physiological correlates (normative data can help picking pictures that are high/low in general involvement)	• comparing affective empathy behaviorally between antisocial PD and controls<br>• identifying affective empathy network in depression (contrasting this condition with a HLB) using PET
E) How strongly do you recognize the following emotions in the scene:<br>• Happiness<br>• Anger<br>• Disgust<br>• ….	Visual analog scale, designed as a slider, ranging from "not at all" to "very much"	Emotional reactivity (basic emotions)<br><br>Emotion recognition	• behavior: individual differences in attitude physiological correlates (normative data can help picking pictures that are high/low in general involvement)	• correlating individual differences in emotional reactivity with personality traits<br>• using individual differences in emotional reactivity as regressor in fMRI contrast of moral judgment
F) What would you do if you were to enter the scene?	Open answer format	Social competence prosociality approach/avoidance	Social approach/avoidance behavior	Training of social competence

Grey = open answers which are not further analyzed in present article

Light blue = MC formats, which for now can just be used as inducing a physiological /brain function, NOT as individual difference measure of performance. This will be done in the near future (because was detailed in script, experts will rate in addition)

White = can be used as inducing a physiological /brain function AND individual difference measure of attitude (behavior)
